# Development and evaluation of the recombinant BP26 protein-based C-ELISA for human brucellosis diagnosis

**DOI:** 10.3389/fmicb.2024.1516915

**Published:** 2025-01-03

**Authors:** Yujia Xie, Shaoqing Lin, Liping Guo, Xinru Qi, Shiqi Zhao, Qichuan Pei, Yixiao Chen, Qi Wu, Yun Wang, Meixue Yao, Dehui Yin

**Affiliations:** ^1^Jiangsu Engineering Research Center of Biological Data Mining and Healthcare Transformation, Xuzhou Medical University, Xuzhou, Jiangsu, China; ^2^Medical Research Center and Clinical Laboratory, Zhuhai People’s Hospital (The Affiliated Hospital of Beijing Institute of Technology, Zhuhai Clinical Medical College of Jinan University), Zhuhai, China; ^3^Department of Dermatology, the Affiliated Huai’an Hospital of Xuzhou Medical University, the Second People’s Hospital of Huai’an, Huai’an, China; ^4^Key Laboratory of Human Genetics and Environmental Medicine, Xuzhou Medical University, Xuzhou, China

**Keywords:** BP26 protein, monoclonal antibody, brucellosis, competitive enzyme-linked immunosorbent assay, diagnosis

## Abstract

**Introduction:**

Timely and accurate diagnosis is crucial for the effective treatment and prevention of brucellosis. Current serological diagnostics, primarily based on lipopolysaccharide (LPS), suffer from cross-reactivity with other Gram-negative bacteria, which limits their specificity. Periplasmic protein 26 (BP26), a highly immunogenic antigen found in *Brucella*, has emerged as a promising alternative for enhancing diagnostic specificity. This study aimed to develop and evaluate a competitive enzyme-linked immunosorbent assay (C-ELISA) utilizing monoclonal antibodies against BP26 for the diagnosis of human brucellosis, thereby providing a more accurate and specific diagnostic approach.

**Methods:**

The study produced monoclonal antibody (mAb) against the BP26 protein through traditional mouse hybridoma technology and developed the C-ELISA method, and compared with a C-ELISA method based on LPS mAb. The detection performance was validated through the analysis of 190 human serum samples, which included 95 brucellosis serum samples and 95 negative serum samples collected by the Xuzhou Center for Disease Control and Prevention, and a comparative analysis was conducted on the diagnostic efficacy of indirect ELISA for brucellosis using both BP26 and LPS-based methods.

**Results:**

The BP26 mAb based C-ELISA achieved 100% sensitivity and specificity in detecting human brucellosis, significantly outperforming the C-ELISA based LPS mAb. Furthermore, the accuracy of the indirect enzyme-linked immunosorbent assay (I-ELISA) using BP26 protein was 98.95%, compared to an accuracy of LPS diagnosis was 99.47%. These results indicated that the BP26 mAb can effectively and accurately detected human brucellosis infections.

**Conclusion:**

This study successfully developed and evaluated a BP26 protein-based C-ELISA method for diagnosing human brucellosis, establishing a foundation for identifying alternative diagnostic antigens for brucellosis.

## 1 Introduction

Brucellosis, a significant zoonotic disease caused by *Brucella* infection, represents a considerable threat to global public health, with an estimated 2.1 million new human cases reported annually worldwide (Laine et al., 2023). In China, the rising number of human cases in recent years, which exceeded 70,000 in 2023, highlights the urgency for effective prevention and control strategies. Timely diagnosis followed by appropriate antibiotic treatment is essential for a favorable prognosis; however, misdiagnosis and delayed treatment can result in chronic infection, often leading poor outcomes (Pappas et al., 2006; Lecároz et al., 2006). Consequently, improving the accuracy of human brucellosis diagnosis is critical for its effective management and control.

Although bacterial culture is regarded as the gold standard for diagnosing brucellosis, its application is limited due to a low positive rate, stringent laboratory safety requirements, and long duration (Raj et al., 2014). Current serological diagnostic methods, such as the standard agglutination test (SAT) and the rose Bengal test, are widely employed; however, they predominantly depend on lipopolysaccharide (LPS) antigens, which exhibit cross-reactivity with other Gram-negative bacteria, thereby limiting their specificity (Eldin et al., 2019). This limitation can lead to misdiagnoses, which has significant repercussions for patient care and disease surveillance. Furthermore, the sensitivity of existing serological techniques remains inadequate. Consequently, the identification of alternative antigens for the diagnosis of brucellosis is a critical endeavor aimed at enhancing both the sensitivity and specificity of serological diagnostics. Previous research has demonstrated that periplasmic protein 26 (BP26), one of the important antigens of *Brucella*, possesses considerable diagnostic value when utilized in the established indirect ELISA method (Yao et al., 2022).

Enzyme-linked immunosorbent assay (ELISA) is a significant serological technique that has been extensively utilized in the clinical diagnosis of various diseases. The competitive enzyme-linked immunosorbent assay (C-ELISA) method is particularly crucial in the serological diagnosis of brucellosis, owing to its high specificity and sensitivity. This study presents a novel application of the C-ELISA method, highlighting its exceptional performance in the detection of human brucellosis infections, characterized by unprecedented levels of sensitivity and specificity.

The purpose of this study was to develop a C-ELISA method utilizing monoclonal antibodies against BP26 protein, and to evaluate its effectiveness in human brucellosis diagnosis, and to provide important scientific evidence for the development of alternative antigens for brucellosis diagnosis.

## 2 Materials and methods

### 2.1 Human serum samples

A total of 190 human serum samples were collected from patients exhibiting fever symptoms, including 95 serum samples from human brucellosis cases and 95 serum samples from individuals who tested negative for brucellosis. The samples from both groups were matched in terms of age, gender, and occupation through individual matching. All serum samples were confirmed by SAT and provided by the Xuzhou Center for Disease Control and Prevention.

### 2.2 Preparation of monoclonal antibodies

Twenty SPF-grade BALB/c female mice, aged 7 weeks and with an average weight of 18 ± 2.0 g, were purchased from Beijing Biotechnology Co., Ltd., (Beijing, China). The mice were housed in cages under a 12-h light/dark cycle and had free access to water and food for 1 week prior to the commencement of the experiment. Four mice were randomly selected and marked for monoclonal antibody preparation, following the procedure below (Guo et al., 2024; Qiu et al., 2012):

For the initial immunization, 60 μg of recombinant BP26 protein (0.5 mg/mL PBS solution, stored in the laboratory) (Bai et al., 2021) was well mixed with an equal volume of Freund’s complete adjuvant (Sigma, USA). The mice were subsequently immunized via multi-point subcutaneous injection. Two weeks later, a booster immunization was conducted using 30 μg of BP26 mixed with an equal volume of Freund’s incomplete adjuvant (Sigma, USA). Booster immunizations were administered biweekly, culminating in a total of five administrations. Following the final booster immunization, serum samples were collected for titer testing. Subsequently, the mice received a final immunization with 50 μg of BP26 via intraperitoneal injection. At the conclusion of the experiment, the mice were euthanized using carbon dioxide. Splenocytes were extracted from the immunized mice and pooled prior to fusion, then mixed with SP2/0 cells at a ratio of 10:1 (splenocytes: SP2/0). PEG1450 (Sigma, Germany) was gradually added to facilitate fusion. After the fusion process, the reaction was terminated using serum-free IMDM (Sigma, Germany), and the precipitate was collected through centrifugation. The precipitate was then resuspended in a culture medium containing 2% HAT and transferred to a 96-well culture plate (100 μL/well, Corning, USA). The plate was incubated at 37? with 5% CO_2_. Five days later, IMDM medium containing 2% HT (Sigma, Germany) and 20% fetal bovine serum (200 μL/well) was added for initial hybridoma screening. Following the screening, BP26 (2 μg/mL) was added, and the culture supernatant was collected. Positive clones were identified through indirect ELISA and subsequently expanded for culture. The mice were injected with 0.5 mL of liquid paraffin. Two weeks later, hybridoma cells at a concentration of 2 × 10^6^/mL were suspended in serum-free medium and injected intraperitoneally (0.5 mL per mouse). Ascitic fluid was collected approximately 8 days later. Monoclonal antibodies present in the ascitic fluid were purified using a protein G column (Sigma, Germany). The same methodology was employed to prepare monoclonal antibodies for LPS (obtained from *Brucella abortus*, 3 mg/mL, gifted by the China Animal Health and Epidemiology Center), with the immunizing dose administered at the same concentration as that of the BP26 protein for immunization purposes.

### 2.3 Identification of BP26-mAb and LPS-mAb

After the purification of the antibodies, they were characterized utilizing 12% SDS-PAGE (Beyotime, China) and Western Blot (WB) (Beyotime, China) kits. In the SDS-PAGE analysis, a loading volume of 10 μg per well was employed for each mAb and antigen. For the Western Blot, the monoclonal antibody was diluted at 1:1000 and the secondary antibody was diluted at 1:5000. The concentration of the mAbs was quantified using the BCA protein assay kit (CWBIO, China). Subsequently, the antibodies were diluted to a concentration of 1 mg/mL, and their subtypes were identified using an indirect ELISA kit (Human ads-UNLB, SouthernBiotech, USA). The characterization of BP26 epitopes recognized by the mAbs was conducted according to previous method (Guo et al., 2024).

The specificity of the BP26-mAb and LPS-mAb was evaluated by indirect ELISA checkerboard titration as fellows: Inactivated whole organisms of *Salmonella* (ATCC 13311), *Escherichia coli* O157:H7 (ATCC 35350), and *Listeria monocytogenes* (LM) (ATCC 19111) were employed as antigens, initially prepared at a concentration of 1 × 10^8^ colony-forming units per milliliter (CFU/mL) in phosphate-buffered saline (PBS). These antigens were coated onto 96-well microtiter plates (Corning, USA) at a dilution of 1:400, followed by a two-fold dilution, and incubated overnight at 4°C. Subsequently, each well was washed three times with 300 μL of PBST (PBS containing 0.05% Tween-20). The BP26 and LPS monoclonal antibodies were then introduced as the primary antibodies, with 100 μL added per well at an initial dilution of 1:200 (5.0 μg/mL) and subjected to a two-fold dilution. The plates were incubated at 37°C for 1 h. Following another three washes with PBST, a horseradish peroxidase (HRP)-conjugated goat anti-mouse IgG secondary antibody (dilution of 1:20,000, Thermo Fisher, USA) was added at a volume of 100 μL per well, and the plates were incubated at 37°C for 30 min. After three additional washes with PBST, 100 μL of TMB substrate solution (100 μg/mL, phosphate-citrate buffer, pH 5.0) was added to each well in the dark for 10 min. The reaction was subsequently halted by adding 50 μL of 2M sulfuric acid (H_2_SO_4_) per well, and the optical density was measured at 450 nm (OD_450_) using a microplate reader (Versa Max microplate reader, MD, USA).

### 2.4 Development of the C-ELISA method

The BP26 protein (0.5 mg/mL) and LPS (3.0 mg/mL) (Yao et al., 2022; Bai et al., 2021) stored in the laboratory were utilized to coat a 96-well microplate (Corning, USA) with Carbonate Buffer Solution (CBS) at a concentration of 10 μg/mL, applying 100 μL per well. The microplate was subsequently incubated at 4°C overnight. Following this incubation, the plate was washed three times with 300 μL of PBST. A blocking solution consisting of 5% skimmed milk was then added to each well at a volume of 300 μL, and the plate was incubated at 37°C for 2 h. After three additional washes with PBST, a mixture of serum and the corresponding monoclonal antibodies (1:1 ratio, diluted in PBS; serum at a final dilution of 1:100; monoclonal antibody at a final dilution of 1:400, 2.5 μg/mL) was introduced to each well at a volume of 100 μL, followed by incubation at 37°C for 1 h. After washing three times with PBST, HRP-conjugated goat anti-mouse IgG secondary antibody (1:20,000 dilution, Thermo Fisher, USA) was added to each well, and the plate was incubated at 37°C for 30 min. Following three washes with PBST, 100 μL of TMB substrate solution was added to each well in the dark for 10 min. The reaction was subsequently halted by adding 50 μL of 2M H_2_SO_4_ per well, and the OD_450_ was measured.

### 2.5 Development of the I-ELISA method

The BP26 protein (0.5 mg/mL) and LPS (3.0 mg/mL) (Yao et al., 2022; Bai et al., 2021) stored in the laboratory were utilized to coat a 96-well microplate at a concentration of 10 μg/mL, 100 μL per well, and the plate was incubated at 4°C overnight. The plate was washed three times with PBST (300 μL per well). Subsequently, 300 μL of 5% skimmed milk blocking solution was added to each well, and the plate was incubated at 37°C for 2 h. After this incubation, the plate was washed three additional times with PBST (300 μL per well). Serum, diluted 1:100 with PBS, was then added at a volume of 100 μL per well and incubated at 37°C for 1 h. The plate underwent three more washes with PBST, after which an HRP- conjugated rabbit anti-human IgG secondary antibody (dilution of 1:20000, Thermo Fisher, USA) was added to each well with 100 μL, incubated at 37°C for 30 min. The plate was washed three times with PBST, and 100 μL of TMB substrate solution each well was added in the dark for 10 min. After stopping the reaction with 50 μL per well of 2M H_2_SO_4_, the OD_450_ was measured.

### 2.6 Evaluation of the detection performance of the C-ELISA and I-ELISA methods

The C-ELISA and I-ELISA methodologies previously established were employed to simultaneously detect the serum samples mentioned above. Each serum sample underwent triplicate testing, and the OD_450_ values were recorded using an ELISA reader, from which the average value was computed. Subsequently, receiver operating characteristic (ROC) curve analysis was conducted to ascertain the cut-off value, and the sensitivity, specificity, and detection concordance rates of the two ELISA methods were evaluated.

### 2.7 Statistical methods

The differences between positive and negative samples were evaluated utilizing an independent samples t-test, conducted with GraphPad Prism version 9.5.0. A *P*-value of less than 0.05 was deemed indicative of a statistically significant difference between the two groups. Furthermore, ROC curve analysis and the generation of scatter plots were executed.

## 3 Results

### 3.1 Preparation of BP26 monoclonal antibodies

**FIGURE 1:**
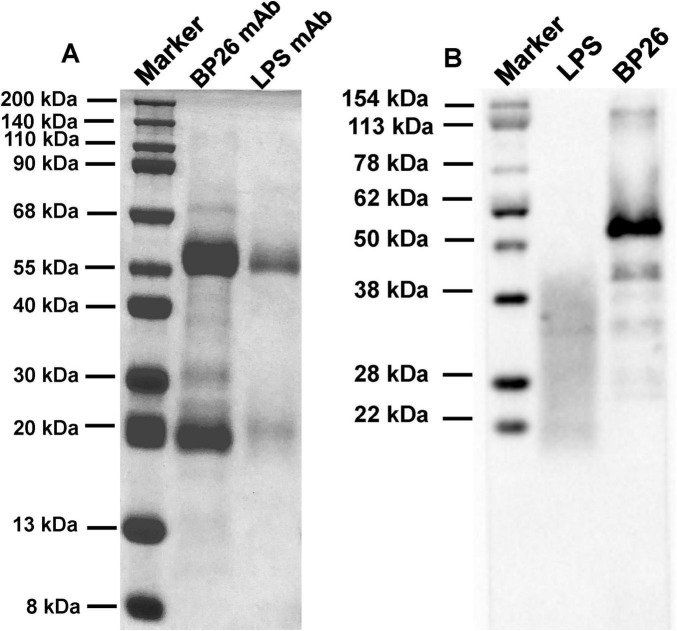
Results of SDS-PAGE and WB for the purified monoclonal antibodies. **(A)** SDS-PAGE of BP26-mAb E10 and LPS-mAb; **(B)** WB of BP26-mAb E10 and LPS-mAb binds to antigens.

Following the screening for cell fusion, a total of 12 BP26 mAbs were successfully expressed. Subsequent identification was performed to assess their peptide recognition capabilities and antibody isotypes (Table 1). Ultimately, the monoclonal antibody designated E10, which specifically recognizes the peptide sequence “QPIYVYPDDKNNLKEPTITGY,” was selected for further analysis. Additionally, only one monoclonal antibody targeting LPS was successfully obtained. These two monoclonal antibodies were utilized to develop the C-ELISA method. The results of the electrophoresis of the purified mAbs, conducted using 12% SDS-PAGE, as well as the Western blot (WB) analysis, are presented in Figure 1. Notably, both BP26-mAb E10 and LPS-mAb exhibited no cross-reactivity with the tested bacterial strains, and the specificity results for BP26-mAb E10 and LPS-mAb are provided in the supporting information.

**TABLE 1:** Subtype of 12 BP26 mAbs.

Named	Antibody subtype							
	**M**	**G1**	**G2a**	**G2b**	**G3**	**A**	**κ**	**λ**
E10	0.070	0.052	0.024	0.886*	0.066	0.091	0.119	0.066
E15	0.065	0.038	0.025	0.590*	0.065	0.061	0.128	0.066
E20	0.069	0.353*	0.029	0.054	0.051	0.061	0.096	0.035
E22	0.067	0.041	0.168*	0.079	0.067	0.062	0.111	0.048
E25	0.066	0.053	0.030	0.606*	0.056	0.071	0.119	0.052
E28	0.082	0.058	0.183*	0.064	0.083	0.085	0.126	0.061
E33	0.076	0.057	0.198*	0.073	0.060	0.082	0.106	0.054
E36	0.070	0.051	0.024	0.809*	0.066	0.075	0.103	0.048
E38	0.065	0.038	0.025	0.714*	0.052	0.060	0.129	0.082
E41	0.049	0.043	0.043	0.733*	0.095	0.068	0.109	0.045
E43	0.058	0.04	0.028	0.541*	0.075	0.102	0.114	0.071
E44	0.064	0.040	0.186*	0.068	0.058	0.087	0.104	0.042
Blank	0.067	0.052	0.027	0.062	0.055	0.095	0.074	0.047

*Positive of antibody subtype.

### 3.2 Detection of serum samples using the C-ELISA method

**FIGURE 2:**
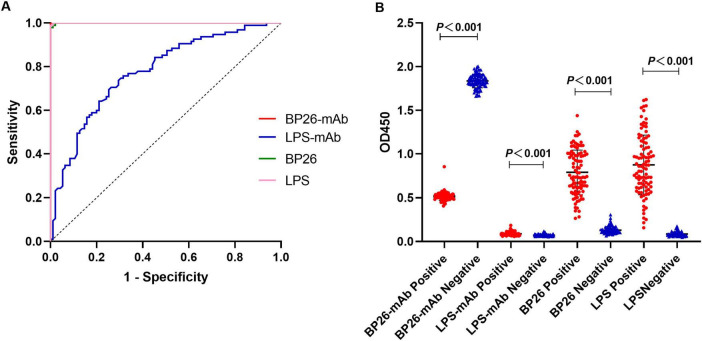
I-ELISA and C-ELISA detection of human serum samples. **(A)** ROC curve for human serum; **(B)** dot plot for human serum.

According to the ROC curve analysis, the area under the diagnostic curve (AUC) for the BP26 mAb-based C-ELISA was 1.000 (95% CI, 1.000∼1.000), and the AUC for the LPS mAb-based C-ELISA was 0.7777 (95% CI, 0.7121∼0.8432), indicating that both have good diagnostic value. Utilizing the Youden index, the diagnostic cut-off value for the BP26 mAb-based C-ELISA was established at 1.257. At this threshold, the sensitivity of the assay was recorded at 1.000 (95% CI, 0.9611–1.000), with a corresponding specificity of 1.000 (95% CI, 0.9611–1.000). Conversely, the cut-off value for the LPS mAb-based C-ELISA was set at 0.0786, yielding a sensitivity of 0.7684 (95% CI, 0.6742–0.8418) and a specificity of 0.6632 (95% CI, 0.5634–0.7502). The detailed results are presented in Figure 2 and Table 2.

**TABLE 2:** Evaluation of ELISA results.

mAb/antigen	Cut-off value	Positive		Negative		Accuracy (%)	PPV (%)	NPV (%)
		**TP**	**FN**	**TN**	**FP**			
BP26-mAb	>1.257	95	0	95	0	100	100	100
LPS-mAb	<0.0786	63	32	73	22	71.58	74.12	69.52
BP26	<0.2744	94	1	94	1	98.95	98.95	98.95
LPS	<0.1953	94	1	95	0	99.47	100	98.96

TP, true positives; TN, true negatives; FP, false positives; FN, false negatives; accuracy, (TP + TN/TP + FN + TN + FP) × 100; PPV, positive predictive value (TP/TP + FP) × 100; NPV, negative predictive value (TN/TN + FN) × 100.

### 3.3 Detection of serum samples using the I-ELISA method

According to the ROC curve analysis, the AUC for the BP26 protein-based I-ELISA was determined to be 0.9997 (95% CI, 0.9989∼1.000), indicating good diagnostic value. Utilizing the Youden index, the diagnostic cut-off value for the BP26 protein-based I-ELISA was established at 0.2744. At this threshold, the sensitivity of the BP26-based I-ELISA was found to be 0.9895 (95% CI, 0.9428–0.9995), while the specificity was also 0.9895 (95% CI, 0.9428–0.9995). Furthermore, the AUC for the LPS-based I-ELISA was calculated to be 0.9999 (95% CI, 0.9995–1.000), indicating similarly strong diagnostic value. The diagnostic cut-off value for the LPS-based I-ELISA, as determined by the Youden index, was 0.1953. At this cut-off value, the sensitivity of the LPS-based I-ELISA was recorded at 1.000 (95% CI, 0.9611–1.000), with a specificity of 0.9895 (95% CI, 0.9428–0.9995). The results are presented in Figure 2 and Table 2.

## 4 Discussion

The *Brucella* outer membrane protein BP26, also known as CP28 or OMP28 (Cloeckaert et al., 1996; Rossetti et al., 1996; Salih-Alj Debbarh et al., 1996), is located on the bacterial surface and within the periplasmic space. It is a soluble protein that can be released from the cell exterior. In comparison to fixed outer membrane proteins, BP26 offers the advantage of enhanced detectability (Salhi et al., 2003; Leclerq et al., 2002). The BP26 protein serves as an effective diagnostic antigen for brucellosis and is capable of distinguishing between natural infections and serological identification of brucellosis following vaccination (Cloeckaert et al., 2001). BP26 is present in all *Brucella* strains, can induce a protective immune response, and demonstrates high immunogenicity in small ruminants, cattle, dogs, and humans, thereby positioning it as a candidate protein for future subunit vaccines (Azizpour Maghvan et al., 2019; Gupta et al., 2019). In China, the approval and implementation of the BP26 mutant vaccine M5ΔBP26 for the prevention of brucellosis in small ruminants underscore the necessity of developing detection methods that target BP26 to facilitate the widespread adoption of the M5ΔBP26 vaccine under the strategy of differentiating infected from vaccinated animals (Li et al., 2017; Zhang et al., 2010). In our previous study, we demonstrated that C-ELISA based on monoclonal antibodies to BP26 could be utilized for the diagnosis of brucellosis in animals and was effective in differentiating between naturally infected and vaccine-immunized (M5ΔBP26) sera (Guo et al., 2024). However, the applicability of this method for the serodiagnosis of human brucellosis has yet to be evaluated.

The existing literature on competitive ELISA methods for brucellosis predominantly emphasizes the detection of the disease in cattle and sheep, with comparatively fewer investigations addressing the application of C-ELISA in human brucellosis. According to the Chinese national standard “Diagnostic Techniques for Brucellosis in Animals” (GB/T 18646—2018), the C-ELISA method is recognized as a serological diagnostic technique for animal brucellosis within China. However, it is noteworthy that the current diagnostic criteria for human brucellosis do not incorporate ELISA as a serological method. The C-ELISA method demonstrates high specificity and sensitivity, rendering it suitable for high-throughput clinical detection of human brucellosis. The incorporation of specific mAbs targeting BP26 in our C-ELISA method significantly diminishes the potential for cross-reactivity with other Gram-negative bacteria, in contrast to assays that depend on LPS antigens. This is attributable to BP26 being a highly immunogenic antigen unique to *Brucella*, which has been shown to exhibit reduced cross-reactivity with other bacterial species. Nonetheless, it is essential to acknowledge that the complete elimination of cross-reactions may necessitate the utilization of alternative antigens alongside the optimization of assay configurations.

As a detection antigen, the BP26 protein exhibits an accuracy exceeding 90%. Consequently, due to its status as a soluble outer membrane protein with high immunogenicity, BP26 is extensively utilized in serological diagnostic research by scholars (Cloeckaert et al., 2001; Wu et al., 2023). This study successfully developed and validated a BP26 mAb based C-ELISA method for diagnosing human brucellosis, which was subsequently compared to an LPS mAb-based C-ELISA. The findings revealed that the BP26 mAbs provided a significant advantage in the detection of brucellosis, particularly regarding sensitivity and specificity. The LPS mAb-based C-ELISA demonstrated lower sensitivity (0.7684) and specificity (0.6632) in comparison to the BP26 mAb-based C-ELISA. With both sensitivity and specificity reaching 100%, the BP26 mAb-based C-ELISA method surpasses conventional LPS-based diagnostics, thereby offering a reliable tool for accurate diagnosis. This suggests that the BP26 protein can serve as a crucial antigen for the diagnosis of brucellosis. The inferior performance of the LPS mAb-based C-ELISA highlights the limitations associated with using LPS as a diagnostic antigen, particularly in regions affected by other Gram-negative infections.

The C-ELISA method is widely acknowledged for its high specificity and sensitivity, particularly in the diagnosis of animal brucellosis (Ameni et al., 2024). In this study, we developed a BP26 mAb-based C-ELISA method and compared its performance with that of the LPS mAb-based C-ELISA, as well as the traditional I-ELISA method. Notably, the accuracy of the LPS-based I-ELISA was determined to be 99.47%. Although this figure is high, it is marginally lower than the accuracy of the BP26-based I-ELISA, which was recorded at 98.95%. This slight discrepancy may be attributed to the superior specificity of the BP26 antigen, which, being a highly immunogenic protein unique to *Brucella*, is less prone to cross-reactivity with other bacterial antigens. The comparative analysis of the diagnostic efficacy of I-ELISA using both BP26 and LPS antigens underscores the advantages of BP26 in serological diagnosis. The significance of this comparison lies in its capacity to validate the potential of BP26 as a diagnostic antigen for brucellosis and to illustrate the benefits of C-ELISA in enhancing diagnostic specificity. By juxtaposing the detection results of C-ELISA and I-ELISA, we can conduct a more comprehensive assessment of the practical application value of the BP26 protein in the diagnosis of brucellosis. This comparison not only facilitates the evaluation of the performance of various diagnostic methods but also equips clinicians with more accurate diagnostic tools, which are essential for the early diagnosis and timely treatment of brucellosis. Furthermore, this analysis provides guidance for future research directions, particularly in the identification and development of new diagnostic antigens for brucellosis. Consequently, the comparison between C-ELISA and I-ELISA is not only significant for this study but also has a substantial impact on the advancement of the entire field of brucellosis diagnosis.

In conclusion, this study demonstrated that the BP26 mAb-based C-ELISA exhibits diagnostic performance comparable to that of the BP26-based I-ELISA utilizing BP26, while also displaying superior specificity in comparison to the LPS-based C-ELISA for the detection of human brucellosis. Furthermore, the findings underscore the significant role of monoclonal antibodies in high-specificity diagnostics, thereby establishing a robust foundation for the exploration of alternative antigens for brucellosis diagnosis. However, the study is not without limitations; specifically, the sample size is relatively small, and not all serum samples were confirmed through bacterial culture. Additionally, there is a lack of comparative analysis with existing diagnostic methods regarding cost-effectiveness, time efficiency, affordability, and other relevant factors. Future research should aim to increase the sample size to further validate the efficacy of the BP26 mAb-based C-ELISA, particularly by incorporating a greater number of random samples.

## Data Availability

The original contributions presented in this study are included in this article/Supplementary material, further inquiries can be directed to the corresponding authors.
